# Fatigue severity in anti-nuclear antibody-positive individuals does not correlate with pro-inflammatory cytokine levels or predict imminent progression to symptomatic disease

**DOI:** 10.1186/s13075-019-2013-9

**Published:** 2019-11-04

**Authors:** Waleed Hafiz, Rawad Nori, Ariana Bregasi, Babak Noamani, Dennisse Bonilla, Larissa Lisnevskaia, Earl Silverman, Arthur A. M. Bookman, Sindhu R. Johnson, Carolina Landolt-Marticorena, Joan Wither

**Affiliations:** 1 Division of Rheumatology, Department of Medicine, Faculty of Medicine, University Health Network and Mount Sinai Hospital, University of Toronto, Toronto, Canada; 2 Division of Genetics and Development, Krembil Research Institute, University Health Network, 5KD402, 60 Leonard Avenue, Toronto, ON M5T 2S8 Canada; 3 Lakeridge Health Services, Oshawa, Canada; 4 Division of Rheumatology, Hospital for Sick Children, Department of Pediatrics, Faculty of Medicine, University of Toronto, Toronto, Canada; 5 Division of Rheumatology, Department of Medicine, Faculty of Medicine, University Health Network, University of Toronto, Toronto, Canada; 6 Institute of Health Policy, Management and Evaluation, University of Toronto, Toronto, Canada; 7 Department of Immunology, Faculty of Medicine, University of Toronto, Toronto, Canada

**Keywords:** Systemic autoimmune rheumatic disease, Fatigue, Cytokines

## Abstract

**Background:**

Fatigue is a common symptom of systemic autoimmune rheumatic disease (SARD). Patients with SARD have a protracted pre-clinical phase during which progressive immunologic derangements occur culminating in disease. In this study, we sought to determine when fatigue develops and whether its presence correlates with inflammatory factors or predicts disease progression.

**Methods:**

Anti-nuclear antibody (ANA)-negative healthy controls (HCs) and ANA-positive participants with no criteria, at least one clinical criteria (undifferentiated connective tissue disease, UCTD), or meeting SARD classification criteria were recruited. Fatigue was assessed using a modified version of the FACIT-F questionnaire and the presence of fibromyalgia determined using a questionnaire based on the modified 2010 ACR criteria. Peripheral blood expression of five IFN-induced genes was quantified by NanoString and the levels of IL-1β, IL-6, or TNF-α by ELISA.

**Results:**

Fatigue was as prevalent and severe in individuals lacking SARD criteria as it was in UCTD and SARD. Overall, ~ 1/3 of ANA^+^ subjects met fibromyalgia criteria, with no differences between sub-groups. Although fatigue was more severe in these individuals, those lacking fibromyalgia remained significantly more fatigued than ANA^−^ HC. However, even in these subjects, fatigue correlated with the widespread pain index and symptom severity scores on the fibromyalgia questionnaire. Fatigue was not associated with elevated cytokine levels in any of the ANA^+^ sub-groups and did not predict imminent disease progression.

**Conclusions:**

Fatigue is common in ANA^+^ individuals lacking sufficient criteria for a SARD diagnosis, correlates with fibromyalgia-related symptoms, and is not associated with inflammation or predictive of disease progression.

## Background

Fatigue is a common feature of the anti-nuclear antibody (ANA)-positive systemic autoimmune rheumatic diseases (SARDs), including systemic lupus erythematosus (SLE), Sjogren’s disease (SjD), systemic sclerosis (SSc), dermatomyositis, and mixed connective tissue disease [1–5]. It can be as disabling as other symptoms of organ dysfunction in these conditions and has a significant negative impact on the quality of life of affected patients [5–9]. Currently, the etiology of fatigue in SARD is poorly understood. Inflammation has been proposed to be a precipitating factor, but a lack of consistent findings showing that fatigue correlates with disease activity or that DMARDs and biologics significantly attenuate fatigue suggests that other factors, such as depression, pain, and poor sleep, contribute to its development [2–4, 6, 9–21].

One of the characteristic features of SARD is a prolonged pre-clinical phase during which autoantibodies are seen in the absence of symptoms [22, 23]. Although this is best established for SLE and SjD, it is likely that this also applies to other SARD. While this suggests that the presence of a positive ANA may predict eventual development of a SARD, ~ 20% of healthy females have a positive ANA [24], the vast majority of which will not progress to SARD. In individuals who progress to a diagnosis of SLE, there is the insidious onset of accumulating clinical symptoms after a variable asymptomatic period [22]. This clinical course is likely also seen in other SARD, since it is not uncommon for individuals to present with insufficient symptoms/signs to classify a SARD (termed undifferentiated connective tissue disease (UCTD)) and positive serologic findings, ~ 20–40% of which go on to develop SARD in the next 3–5 years [25–27].

Studies suggest that as patients progress from no symptoms to a diagnosis of SARD, there is a progressive increase in the levels as well as a change in the types of pro-inflammatory cytokines that are elaborated [28–30]. Given the proposed link between inflammation and fatigue, physicians are often concerned that the presence of profound fatigue in ANA^+^ individuals may indicate the presence of unappreciated inflammation and a consequent increased risk of progression. In this study, we have addressed this question by examining fatigue in individuals who span the ANA^+^ disease continuum from asymptomatic through UCTD to early SARD.

## Methods

### Subjects and data collection

ANA^+^ individuals (≥ 1:160 by immunofluorescence), who had been referred to a clinic because of a recently discovered positive ANA, were consecutively recruited at the Toronto Western and Mount Sinai Hospitals. All patients were assessed by at least one of the participating rheumatologists and followed prospectively with clinical data being recorded through the use of standardized data collection forms. Participants were stratified into three groups based upon their initial assessment: (1) asymptomatic individuals, who lacked any clinical symptoms of SARD; (2) UCTD patients that had at least one clinical symptom of SARD but insufficient criteria to be classified as SARD; and (3) early SARD patients meeting classification criteria for a SARD (1997 ACR classification criteria for SLE [31], 2013 ACR-EULAR classification criteria for SSc [32], or the revised American-European consensus criteria for SjD [33]) and that were within 2 years of diagnosis (except for SjD < 5 years). Subjects were excluded if they were on corticosteroids or DMARDS (except anti-malarials). All healthy controls (HCs) had their ANA and specific autoantibodies tested in the hospital laboratory to confirm that they were negative. HC with an ANA ≥ 1:160 were re-classified into the asymptomatic ANA^+^ group, and those with a positive ANA < 1:160 or specific ANAs were excluded from the study. The study was approved by the Research Ethics Boards of both recruiting hospitals, and all participants signed informed consent.

### Clinical measures

Fatigue was quantified using a modified version of the Functional Assessment Chronic Illness Therapy—Fatigue (FACIT-F) questionnaire with two questions that potentially might apply to disability rather than fatigue and one question regarding sleepiness in the day, a potential symptom of fibromyalgia, being removed [34]. To permit comparison with other studies using the FACIT-F, the score was calculated as 13 (the original number of questions on the FACIT-F) × the total score for answered questions divided by the number of questions answered. The presence of fibromyalgia was determined using a self-reported questionnaire using the modified 2010 ACR criteria [35, 36]. Patients were defined as having anemia if their hemoglobin level < 115 g/L, hypothyroidism if their TSH > 5.5 mU/L and free T4 < 11 pmol/L, and depression if they were diagnosed by a physician and were on anti-depressant therapy.

### Measurement of autoantibodies

Autoantibodies were measured in the University Health Network laboratory, with the ANA titer and pattern being determined by indirect immunofluorescence using HEp-2 cells as a substrate. The levels of 11 specific autoantibodies (dsDNA, -chromatin, -Ro, -La, -Sm, -SmRNP, -RNP, -Jo-1, -Scl-70, -centromere, and ribosomal P) were assayed by the Bioplex® 2200 ANA Screening System (BioRad), using the company’s cutoffs.

### Cytokine measurement

For measurement of interferon (IFN)-induced gene expression, total RNA was isolated from whole peripheral blood archived in Tempus tubes (Applied Biosystems) and gene expression was quantified by NanoString using a custom array (nanoString Technologies), as previously described [24]. Log_2_ normalized expression levels of five IFN-induced genes (*EPSTI1*, *IFI44L*, *LY6E*, *OAS3*, *RSAD2*) were summed to generate a composite IFN5 score. Serum IFN-α and BAFF levels were measured by ELISA, as previously described [24], and serum IL-1-β, IL-6, and TNF-α levels using Quantikine High Sensitivity ELISA kits (R&D Systems).

### Statistical analysis

For comparisons of differences between three or more groups, a Kruskal-Wallis test was used followed by Dunns’ post-test for multiple comparisons. When only two groups were compared, the Mann-Whiney *U* test was performed for continuous variables and a *χ*^2^ or Fisher’s exact test for discrete variables. The significance of association between variables was determined using Spearman’s correlation coefficient. All statistical analyses were performed using GraphPad software (La Jolla, CA, USA).

## Results

### Fatigue is commonly seen in ANA^+^ individuals lacking a SARD diagnosis and is often associated with symptoms of fibromyalgia

Demographics for the 146 study participants are summarized in Table 1. The majority of participants were female. Although the range of ages in each group was similar, the mean age for HCs was significantly lower than that for the three ANA^+^ sub-groups (ANA^+^ no SARD symptoms (ANS), UCTD, SARD). Overall, 58% of participants were Caucasian with a non-significant trend to fewer Caucasians in the HC group. A small number of ANA^+^ participants were taking anti-malarials, including four individuals with ANS who had been started on anti-malarials prior to assessment in the clinic for symptoms that could not be definitely attributed to SARD (myalgia, arthralgia, and fatigue).

**Table 1:** Study participant characteristics

	HC (*n* = 29)	ANS (*n* = 46)	UCTD (*n* = 29)	SARD (*n* = 42)	SjD (*n* = 11)	SLE (*n* = 11)	SSc (*n* = 18)	MCTD/DM (*n* = 2)
Age, mean ± SD	29.3 ± 9.8	47.0 ± 14.0	48.3 ± 16.5	47.4 ± 14.9	47.7 ± 14.4	35.7 ± 12.3	54.7 ± 13.3	44 ± 1.4
Female sex, *n* (%)	25 (86.2)	44 (95.7)	27 (93.1)	40 (95.2)	10 (90.9)	11 (100)	16 (88.9)	2 (100)
Ethnicity, *n* (%)								
Caucasian	12 (41.4)	26 (56.5)	20 (69.0)	26 (61.9)	7 (63.6)	6 (54.5)	12 (66.7)	1 (50)
Asian	0 (0)	3 (6.5)	5 (17.2)	2 (4.8)	1 (9.1)	0 (0)	1 (5.6)	0 (0)
South Asian	5 (17.2)	5 (10.9)	2 (6.9)	5 (11.9)	2 (18.2)	1 (9.1)	2 (11.1)	0 (0)
Hispanic	7 (24.1)	2 (4.3)	1 (3.4)	4 (9.5)	0 (0)	1 (9.1)	3 (16.7)	0 (0)
African Canadian	1 (3.4)	7 (15.2)	0 (0)	1 (2.4)	0 (0)	1 (9.1)	0 (0)	0 (0)
Filipino	1 (3.4)	1 (2.2)	0 (0)	2 (4.8)	0 (0)	1 (9.1)	0 (0)	1 (50)
Mixed	3 (10.3)	2 (4.3)	1 (3.4)	2 (4.8)	1 (9.1)	1 (9.1)	0 (0)	0 (0)
Fibromyalgia, *n* (%)	0 (0)	17 (37.0)	13 (44.8)	12 (28.6)	2 (18.2)	3 (27.3)	6 (33.3)	1 (50.0)
Anemia, *n* (%)	0 (0)	4 (8.7)	0 (0)	2 (4.8)	0 (0)	1 (9.1)	1 (5.6)	0 (0)
Hypothyroidism, *n* (%)	0 (0)	4 (8.7)	0 (0)	2 (4.8)	1 (9.1)	0 (0)	1 (5.6)	0 (0)
Depression, *n* (%)	0 (0)	3 (6.5)	2 (6.9)	2 (4.8)	1 (9.1)	0 (0)	1 (5.6)	0 (0)
On anti-malarials, *n* (%)	0 (0)	4 (8.7)	6 (20.7)	4 (9.5)	1 (9.1)	2 (18.2)	1 (5.6)	0 (0)
Specific antibodies, *n* (%)								
dsDNA	0 (0)	4 (8.7)	2 (6.9)	7 (16.7)	2 (18.2)	3 (27.3)	2 (11.1)	0 (0)
Ro	0 (0)	11 (23.9)	9 (31.0)	19 (45.2)	11 (100)	5 (45.5)	3 (16.7)	0 (0)
La	0 (0)	4 (8.7)	2 (6.9)	8 (19.0)	7 (63.6)	1 (9.1)	0 (0)	0 (0)
Sm	0 (0)	2 (4.3)	1 (3.4)	4 (9.5)	0 (0)	3 (27.3)	0 (0)	1 (50.0)
Sm/RNP	0 (0)	3 (6.5)	2 (6.9)	6 (14.3)	0 (0)	4 (36.4)	1 (5.6)	1 (50.0)
RNP	0 (0)	6 (13.0)	3 (10.3)	8 (19.0)	2 (18.2)	4 (36.4)	1 (5.6)	1 (50.0)
Scl-70	0 (0)	1 (2.2)	1 (3.4)	8 (19.0)	1 (9.1)	2 (18.2)	5 (27.8)	0 (0)
Jo-1	0 (0)	0 (0)	0 (0)	0 (0)	0 (0)	0 (0)	0 (0)	0 (0)
Centromere	0 (0)	1 (2.2)	3 (10.3)	15 (35.7)	0 (0)	1 (9.1)	13 (72.2)	1 (50.0)
Chromatin	0 (0)	5 (10.9)	2 (6.9)	7 (16.7)	1 (9.1)	5 (45.5)	0 (0)	1 (50.0)

*Abbreviations*: *HC* healthy controls, *ANS* asymptomatic ANA^+^, *UCTD* undifferentiated connective tissue disease, *SARD* systemic autoimmune rheumatic disease, *SjD* Sjögren’s disease, *SLE* systemic lupus erythematosus, *SSc* systemic sclerosis, *MCTD* mixed connective tissue disease, *DM* dermatomyositis, *dsDNA* double-stranded DNA, *Sm* Smith, *RNP* ribonuclear protein

**Fig. 1:**
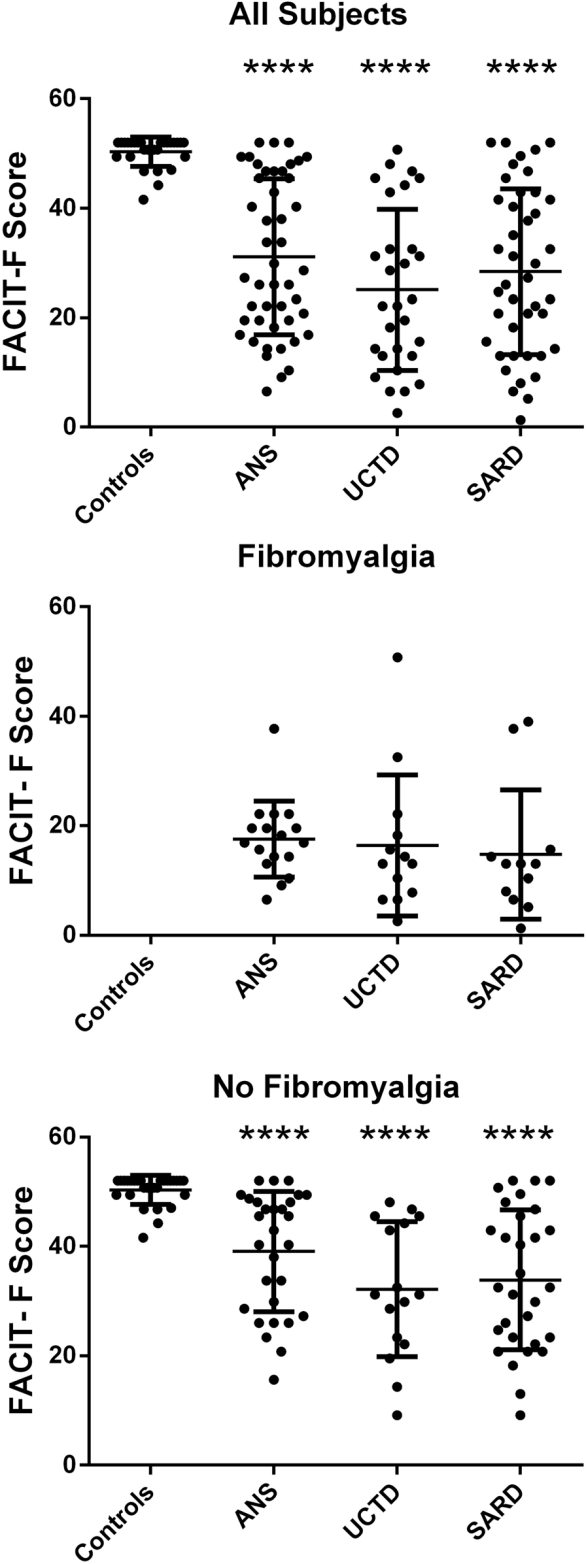
Asymptomatic ANA^+^ individuals lacking a SARD diagnosis have just as severe fatigue as UCTD and SARD patients. Shown are scatterplots with results for all subjects and subdivided into those with and without a diagnosis of fibromyalgia. Columns indicate results for ANA^−^ healthy controls (Controls), asymptomatic ANA^+^ individuals (ANS), and patients with UCTD or SARD. Every data point corresponds to an individual subject, with the bars representing the mean with SD. For each set of comparisons, statistical significance was determined using the Kruskal-Wallis test with Dunn’s post-test for multiple comparisons, as compared to controls. *p ≤ 0.05, **p ≤ 0.01, ***p ≤ 0.001, ****p ≤ 0.0001. There were no significant differences between the different ANA^+^ sub-groups

The presence of fatigue was determined using a modified version of the FACIT-F questionnaire, where lower scores indicate the presence of more fatigue. As shown in Fig. 1, all ANA^+^ subjects regardless of the presence (SARD and UCTD) or absence of SARD symptoms/criteria (ANS) were significantly more fatigued than HCs, with no significant differences noted between the different ANA^+^ sub-groups in the extent of fatigue. Using a cutoff of 3 SD below the mean for ANA^−^ HC as significant fatigue, 67.4% of ANS, 79.3% UCTD, and 80.9% of SARD subjects were fatigued, as compared to 3.4% of ANA^−^ HC. Because many of the subjects suffered from fibromyalgia, and indeed this may have led to ANA testing in the case of ANS, we examined whether the fatigue was related to fibromyalgia, using the modified 2010 ACR criteria [35]. Individuals with a widespread pain index (WPI) of ≥ 7 and a symptom severity (SS) score of ≥ 5, or a WPI between 3 and 6 and a SS score ≥ 9, on a self-administered questionnaire were considered to have fibromyalgia, which has been shown to have a sensitivity of 96.6% and specificity 91.8% for patients diagnosed clinically with fibromyalgia. Using this cutoff, none of the healthy controls and 37% of the ANA^+^ subjects had fibromyalgia (*p* < 0.0001), with similar proportions of patients with fibromyalgia in each of the three ANA^+^ sub-groups (see Table 1). Not surprisingly, the FACIT-F scores were significantly lower in patients with fibromyalgia as compared to those without fibromyalgia and this was the case not only for the ANA^+^ subjects as a whole (mean FACIT-F ± SD, 35.5 ± 12.2 without fibromyalgia, 16.4 ± 10.3 with fibromyalgia, *p* < 0.0001) but also for each of the ANA^+^ sub-groups (*p* < 0.0001, except UCTD *p* = 0.0026) (Fig. 1). However, ANA^+^ individuals without fibromyalgia still had significantly lower FACIT-F scores as compared to HC (*p* < 0.0001), and again this remained true for each of the ANA^+^ sub-groups (Fig. 1).

**Fig. 2:**
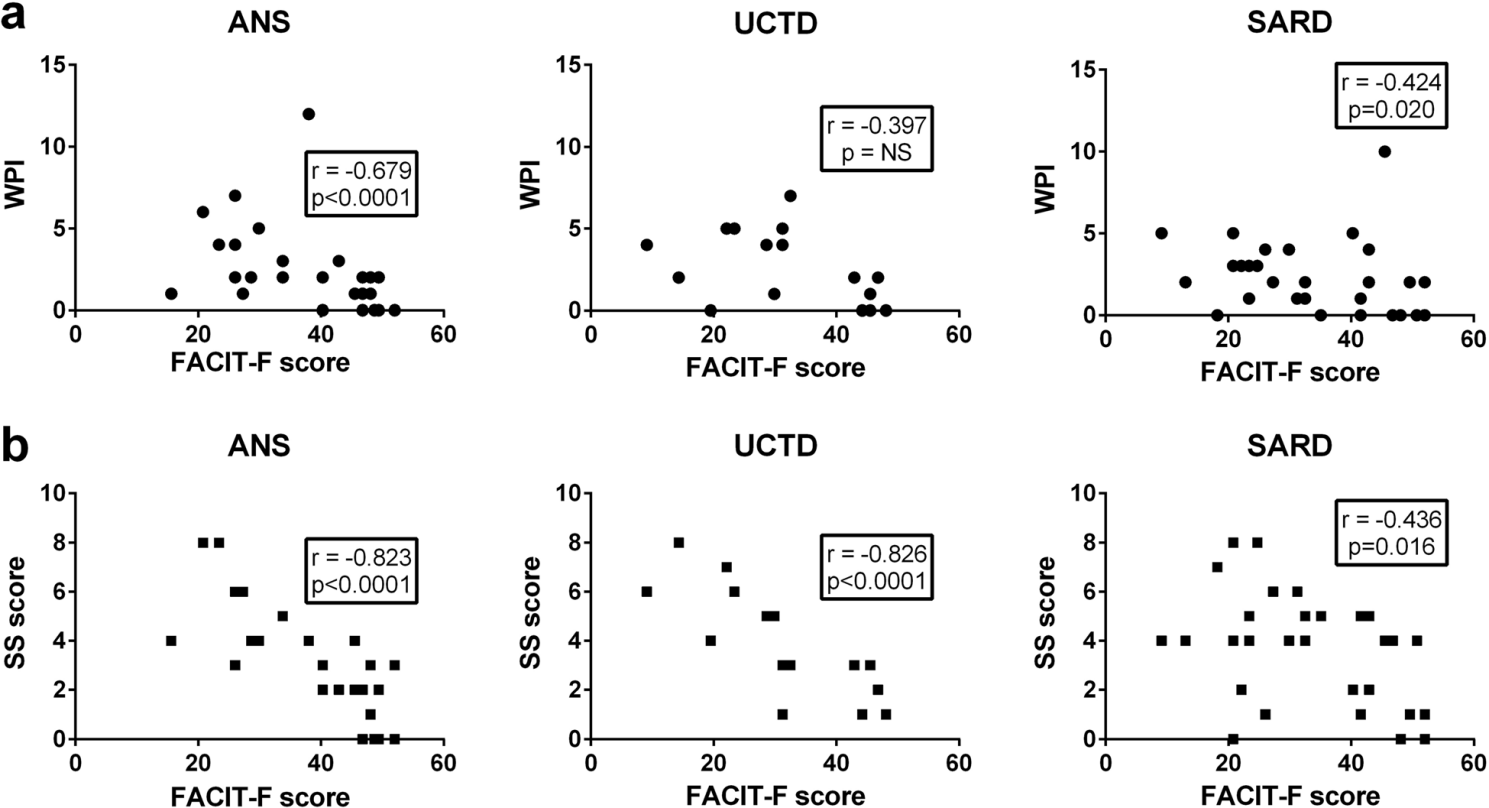
Correlations between fatigue and the widespread pain index (WPI) or symptom severity (SS) scores on the fibromyalgia questionnaire for the subjects without a fibromyalgia diagnosis. Results have been stratified into the different ANA^+^ sub-groups (asymptomatic ANA^+^ individuals (ANS), UCTD, and SARD). **a** Correlation between the WPI score and FACIT-F score. **b** Correlation between the SS score and FACIT-F score. Every symbol corresponds to an individual subject. Values in the boxes show the Spearman correlation coefficient and significance of association

The WPI and SS scores derived from the fibromyalgia questionnaire represent a continuum that reflects the extent of pain and fatigue/somatic symptoms, respectively, independently of a diagnosis of fibromyalgia [36]. We therefore questioned whether the FACIT-F score correlated with these scores, even in the absence of fibromyalgia. As shown in Fig. 2, there was a strong negative correlation between the WPI and SS scores and the FACIT-F score in ANS, suggesting that the fatigue in these individuals may be related to symptoms of fibromyalgia. Of note, this was not simply due to redundancy between the questions being asked in the two questionnaires because only the SS score partially overlaps with the FACIT-F questionnaire, and equivalent strong correlations were seen for both WPI and SS sub-components. While the correlations between the FACIT-F score and WPI and SS scores were somewhat weaker in UCTD and SARD patients lacking a fibromyalgia diagnosis (Fig. 2), there remained moderate negative correlations with the FACIT-F score, suggesting that in these patients as well a component of the fatigue may be due to fibromyalgia-related symptoms.

**Fig. 3:**
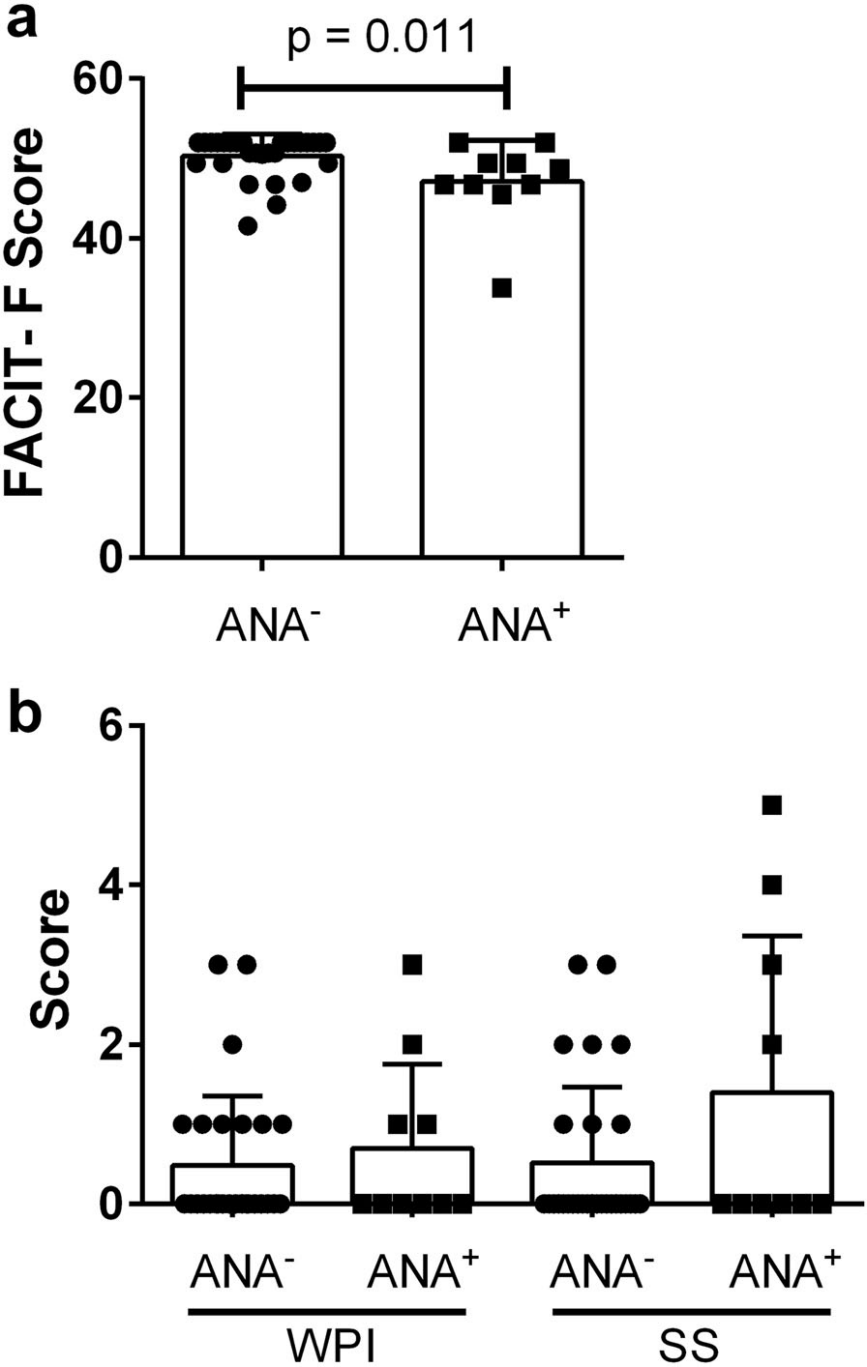
Presence of mild fatigue in ANA^+^ individuals who were recruited as healthy controls or who gave birth to a baby with neonatal lupus. **a** Fatigue, as measured by the FACIT-F score, and **b** WPI and SS scores, as measured by the fibromyalgia questionnaire, in ANA^−^ healthy controls (ANA^−^) and ANA^+^ individuals, as outlined above (ANA^+^). Every symbol corresponds to an individual subject with bars indicating the mean with SD. Significant differences are shown and were calculated using the Mann-Whitney *U* test comparing ANA^−^ and ANA^+^ subjects

To further explore whether the fatigue in ANS individuals is predominantly related to symptoms of fibromyalgia, we compared the FACIT-F scores in the subset of ANA^+^ subjects without SARD symptoms that had been recruited solely based upon their positive serology with those for HCs. These subjects included anti-Ro antibody-positive mothers who were referred for longitudinal follow-up after giving birth to a child with neonatal lupus or congenital heart block, and healthy controls re-classified to the ANS group following discovery of a positive ANA (≥ 1:160) on laboratory testing. Nine subjects fulfilled these criteria, none of whom fulfilled criteria for fibromyalgia. As shown in Fig. 3, the FACIT-F scores for these subjects were significantly lower than those for the ANA^−^ HCs, despite WPI and SS scores that were roughly equivalent to HCs. This finding suggests that fatigue may be associated with a positive ANA and in support of this possibility an additional subject who was recruited as a HC, who was found to have anti-Ro Abs but did not meet study criteria for inclusion in the ANA^+^ subset, also had a low FACIT-F score (FACIT-F = 27.3). However, the impact of a positive ANA on fatigue appeared to be quite modest as compared to that of fibromyalgia-type symptoms.

As comorbidities, such as anemia, hypothyroidism, or depression, have been shown to contribute to chronic fatigue [34, 37–39], we assessed whether fatigue was more profound in ANA^+^ subjects with these diagnoses. Very few of the subjects had these comorbidities (Table 1), and no significant differences were seen in the FACIT-F scores between subjects with and without these conditions (data not shown, all *p* > 0.05).

### The presence of fatigue does not correlate with inflammation in any of the ANA^+^ sub-groups

Consistent with the possibility that fatigue in SARD results from inflammation, some studies have found a correlation with disease activity and/or reductions in fatigue following treatment with DMARDs or biologics [2, 11, 15, 17, 18, 21]. Of the pro-inflammatory cytokines that are typically elevated in SARD, IL-1β, IL-6, and TNF-α, in particular, have been linked to fatigue [40–42].

**Fig. 4:**
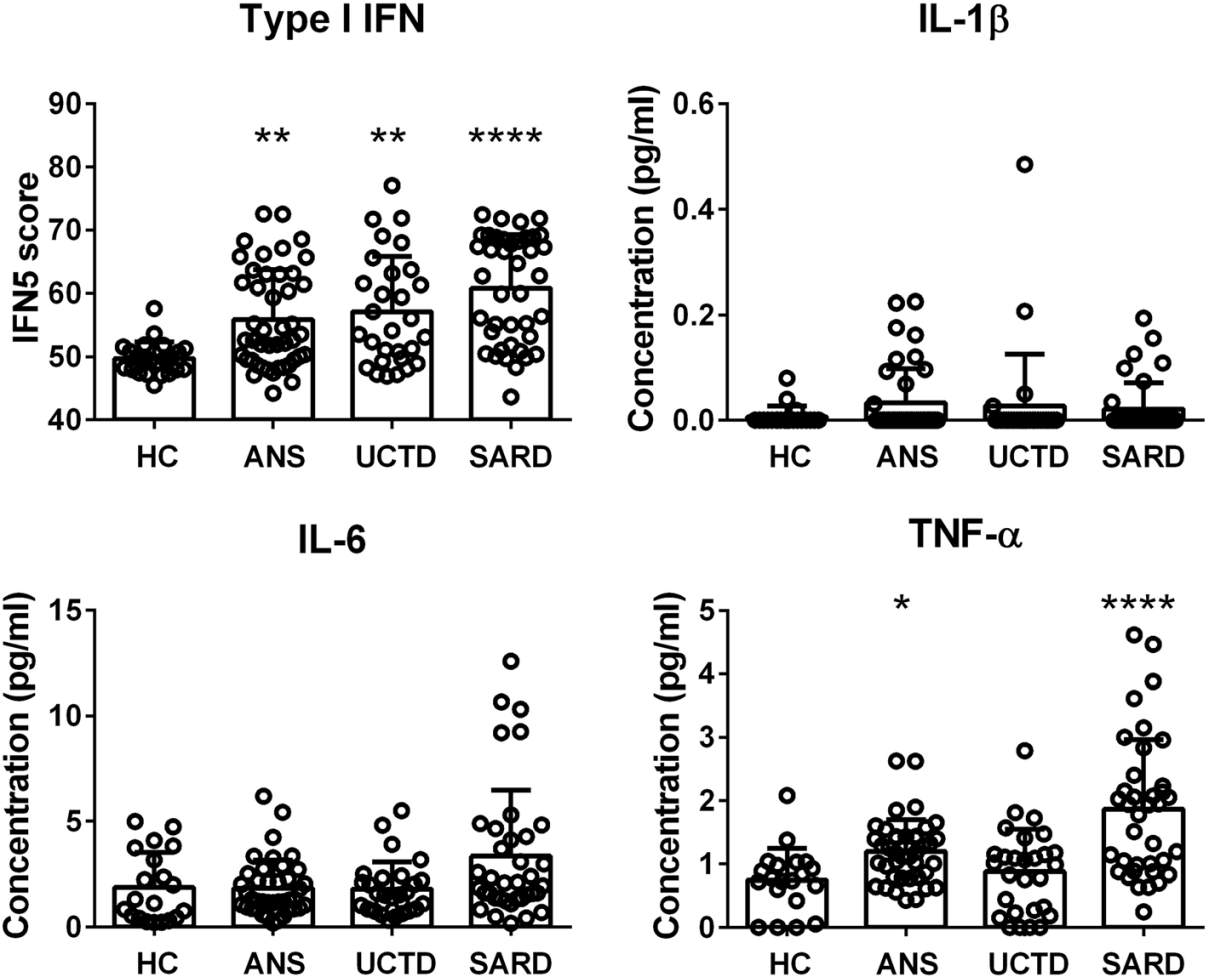
Levels of selected pro-inflammatory cytokines in ANA^+^ individuals stratified based upon the presence of clinical SARD diagnostic criteria. Columns indicate results for ANA^−^ healthy controls (HC), ANA^+^ individuals lacking any SARD clinical diagnostic criteria (ANS), and patients with UCTD or SARD. Every data point corresponds to an individual subject, with the bars representing the mean with SD. For each set of comparisons, statistical significance was determined using the Kruskal-Wallis test with Dunn’s post-test for multiple comparisons, as compared to HC. *p ≤ 0.05, **p ≤ 0.01, ***p ≤ 0.001, ****p ≤ 0.0001

As outlined previously, there was no association between the FACIT-F score and the presence or absence of SARD symptoms/signs in ANA^+^ subjects (see Fig. 1) nor was there an association between ANA titer or the number of different ANA specificities as measured by the Bioplex ANA screen and fatigue (data not shown). To examine the association between fatigue and inflammation, we quantified the levels of type I IFN-induced gene expression as well as the serum levels of IL-1β, IL-6, and TNF-α. We have previously shown that a significant proportion of ANA^+^ individuals have elevated type I IFN levels including those without SARD symptoms/signs and that these elevations correlate with the levels of several IFN-driven cyto/chemokines, such as BAFF [24]. Similar elevations of IFN-induced gene expression were seen in the ANA^+^ individuals that were examined in this study (some of which overlapped with those previously published, Fig. 4), which did not correlate with fatigue (Table 2). As IL-1β was not significantly elevated in any of the ANA^+^ groups when compared to HC, and given that the levels of IL-β in > 50% of the samples were below the limit of detection of the ELISA, associations with this cytokine were not examined further. A trend to increased levels of IL-6 and TNF-α was seen in all ANA^+^ groups as compared to HC, which was most pronounced in SARD. This achieved statistical significance only for TNF-α in ANS and SARD patients. As shown in Table 2, there was no association between fatigue and any of the cytokines, either for ANA^+^ individuals as a whole or for any of the ANA^+^ sub-groups, and similar negative findings were seen when patients with and without fibromyalgia were examined independently (Additional file 1: Table S1).

**Table 2:** Associations with inflammatory cytokines

Cytokine	All ANA+, *ρ*	ANS, *ρ*	UCTD, *ρ*	SARD, *ρ*
A. FACIT-F				
Type I IFN	0.071	0.118	0.213	− 0.029
IL-6	− 0.072	− 0.092	− 0.224	0.015
TNF-α	− 0.101	− 0.093	− 0.135	− 0.152
B. WPI				
Type I IFN	− 0.064	− 0.142	− 0.038	− 0.022
IL-6	0.239 (0.014)*	0.308 (0.053)	0.211	0.288 (0.084)
TNF-α	0.090	0.258	0.083	0.201
C. SS score				
Type I IFN	− 0.164 (0.081)	− 0.132	− 0.204	− 0.076
IL-6	0.174 (0.075)	0.223	*0.397 (0.036)*	0.090
TNF-α	− 0.005	0.089	0.057	0.011

*Significant differences are indicated in italics with the *p* value shown in the brackets. Any *p* values less than 0.1 are also shown as indicative of trends

To further explore the association between fibromyalgia, pain, fatigue, and inflammation, we examined the correlation between cytokine levels and the presence or absence of fibromyalgia, as well as the WPI and SS scores. There were no significant differences in the levels of cytokines between ANA^+^ individuals with or without at fibromyalgia diagnosis (data not shown). However, there was a significant correlation between IL-6 levels and the WPI for the ANA^+^ individuals as a whole, which remained marginally significant in the ANS and SARD sub-groups (Table 2). In general, the strength of this association was greater than that seen for IL-6 levels with the SS score (with the exception of the UCTD sub-group, see Table 2) and also was greater for individuals without a fibromyalgia diagnosis as compared to those with a fibromyalgia diagnosis (Additional file 1: Table S2). These findings suggest that the association of IL-6 levels with WPI may be independent of fatigue and instead may indicate that a component of the arthralgia results from inflammation. In support of this concept, there was also a significant association between TNF-α levels and the WPI in ANA^+^ subjects without fibromyalgia, which was largely driven by the SARD sub-group.

### Severe fatigue does not predict imminent progression to SARD

Physicians are often concerned that the presence of profound fatigue in ANA^+^ individuals might indicate an increased likelihood of progression to a UCTD or SARD. To investigate this possibility, we contrasted fatigue in patients who demonstrated progression, as indicated by the development of new SARD classification criteria, as compared to those who did not. There were 3 of the 26 ANS patients with at least 1 year of follow-up who developed definitive SARD criteria (1 developed seropositive rheumatoid arthritis, 1 had the development of new lupus-associated autoantibodies and Raynaud’s phenomenon, and 1 developed arthritis, rash, and Raynaud’s phenomenon fulfilling classification criteria for SLE). There was a non-statistically significant trend to less fatigue in progressors compared to non-progressors (median FACIT-F: progressors 46.8, non-progressors 26, *p* = 0.150). Decreases were also seen in the WPI and SS scores for progressors, which achieved statistical significance for the SS score (*p* = 0.031). Consistent with a recently published study suggesting that ANA^+^ individuals lacking a SARD diagnosis with high IFN scores are more likely to progress to SARD than those with low IFN scores, the IFN scores in progressors were significantly higher than in non-progressors (*p* = 0.0054). No differences were seen in the levels of IL-6 and TNF-a between progressors and non-progressors.

Similar but less pronounced findings were observed for patients with UCTD. Four of 22 UCTD patients progressed in a 1-year follow-up period, with development of new SARD criteria (1 new onset arthritis) or evolution to SARD (2 SjD, 1 SSc). Although there were trends to decreased fatigue, WPI, and SS scores, as well as increased IFN scores in progressors as compared to non-progressors, these did not achieve statistical significance.

## Discussion

In this study, we show that the prevalence and severity of fatigue in ANS is similar to that seen in UCTD and early SARD and comparable to that seen in previous studies of ANA^+^ SARD where the FACIT-F was used to quantify fatigue [8, 9, 43]. As noted in other studies of SARD, a substantial component of this fatigue was related to fibromyalgia [44–46], which was present in ~ 1/3 of all ANA^+^ subjects regardless of the presence or absence of SARD criteria, and which was associated with significantly more marked fatigue as measured by the FACIT-F than seen in subjects lacking fibromyalgia. Nevertheless, even ANA^+^ subjects lacking fibromyalgia were still significantly more fatigued than ANA^−^ HC and the severity of the fatigue was again similar in ANS to that observed for UCTD and SARD patients.

In ANS lacking fibromyalgia, there remained a strong correlation between the WPI and SS scores and the FACIT-F, suggesting that although these patients did not meet criteria they may still have had fibromyalgia-like symptoms. This may reflect a selection bias, where individuals with pain and fatigue are more likely to seek medical care and have serologic testing performed, in part due to the perception that these symptoms may be a surrogate for ongoing inflammation. However, similar but slightly weaker correlations were also seen for UCTD and early SARD patients, indicating that even in individuals who have SARD criteria, a significant component of their fatigue may be due to fibromyalgia-like symptoms. The findings are in keeping with previous studies showing a correlation between fibromyalgia, disturbances of sleep, tender points, or pain and fatigue in SARD [6, 16, 45, 46].

We recognize that the diagnostic criteria that we used for fibromyalgia were developed and validated for patients without inflammatory rheumatic disease. However, we used these in UCTD and SARD patients to enable comparison with ANA^−^ HC and ANS subjects and because the majority of our patients lacked inflammatory arthritis. Of the 12 SARD patients that met diagnostic criteria for fibromyalgia, only 3 had tender joints thought to be related to inflammatory arthritis, with only one having swollen joints. Notably, all 3 of these patients had more generalized pain on their fibromyalgia questionnaire than could be accounted for by their tender joints. In fact, the majority of SARD patients (9/12) that met fibromyalgia criteria had a WPI ≥ 7. Thus, SARD patients did not solely meet fibromyalgia criteria based upon their fatigue symptoms, but also had substantial unexplained generalized pain consistent with this diagnosis. These findings are compatible with previous studies that found an increased prevalence of fibromyalgia in SARD patients, either when diagnosed using conventional clinical criteria or the diagnostic criteria used in this study [47–50].

The close correlation between fatigue and fibromyalgia-like symptoms in ANA^+^ individuals that are referred to a rheumatologist lacking clinical SARD diagnostic criteria made it difficult to assess whether the presence of an ANA alone was associated with fatigue. We circumvented this problem by examining ANS who had been recruited as HC or whose ANA was discovered following delivery of a baby with neonatal lupus. Although fibromyalgia-like symptoms in these individuals were no more prevalent than in ANA^−^ HC, they were statistically significantly more fatigued. However, the magnitude of this difference was small and the severity of this fatigue was very mild, suggesting that the majority of the fatigue seen in the ANS individuals referred to rheumatologists is unrelated to the immunologic derangement that produces a positive ANA. In support of this concept, no correlation was seen between ANA titer or number of different ANA specificities and the extent of fatigue.

The contribution of inflammation to fatigue in rheumatic diseases remains unclear. Studies showing that injection of some of the key cytokines produced in rheumatic diseases into HC, such as IL-1β or IL-6, produces fatigue and that biologics targeting IL-6 or TNF-α ameliorate fatigue [11, 40–42] suggest a role for these molecules in the development of fatigue. However, while the levels of these cytokines tend to correlate with disease activity, very few studies have shown an association between disease activity and fatigue [2, 15, 17]. Furthermore, where these cytokines have been measured, no correlation has been noted [14, 19, 20]. In this study, we show that although the levels of TNF-α are significantly elevated in SARD and ANS, and there is a trend to increased IL-6 in these groups, there was no correlation with fatigue, confirming previous studies of SARD [6, 10, 12, 16] and indicating that this extends to individuals with ANS and UCTD. However, some correlations were seen for these cytokines with the WPI. Since the majority of patients did not suffer from joint inflammation even within the SARD group, it is possible that these associations reflect the ability of IL-6 and TNF-α to stimulate nociceptive sensory neurons leading to enhanced pain sensitivity [51].

We and others have previously shown that elevated levels of type I IFN are associated with symptomatic progression in ANS and UCTD [52, 53]. Here, we show that there is no association between type I IFN levels and fatigue and that fatigue does not predict symptomatic progression. Indeed, there was a non-significant trend to less fatigue in progressors.

## Conclusions

Our findings have important clinical implications. Firstly, clinicians can reassure their fatigued ANS patients that their fatigue does not indicate that they are at increased risk for imminent progression; secondly, the presence of significant fatigue should not prompt initiation of treatment with DMARDs; and thirdly, our findings suggest that treatments that have been shown to improve fatigue, such as exercise programs, promotion of good sleep hygiene, addressing life stressors and depression [54], or drug therapy for fibromyalgia, may be more appropriate therapies for these individuals.

## Supplementary information

**Additional file 1: Table S1.** Correlations between the FACIT-F score and inflammatory cytokines in ANA^+^ subjects. **Table S2.** Correlations between the WPI and inflammatory cytokines in ANA^+^ subjects.

## Data Availability

Supporting data is located in Additional file 1.

## Abbreviations

ANA: Anti-nuclear antibody
SARD: Systemic autoimmune rheumatic disease
SLE: Systemic lupus erythematosus
SjD: Sjogren’s disease
SSc: Systemic sclerosis
UCTD: Undifferentiated connective tissue disease
HC: Healthy control
FACIT-F: Functional Assessment Chronic Illness Therapy-Fatigue
IFN: Interferon
ANS: ANA^+^ no SARD symptoms
WPI: Widespread pain index
SS: Symptom severity
