# Supplementary material for: Fatigue severity in anti-nuclear antibody-positive individuals does not correlate with pro-inflammatory cytokine levels or predict imminent progression to symptomatic disease
Source: Arthritis Res Ther. 2019 Nov 4;21:223. doi: 10.1186/s13075-019-2013-9 (PMC6827224; doi:10.1186/s13075-019-2013-9)
Supplement: Supplementary file 1 — Additional file 1: Table S1. Correlations between the FACIT-F score and inflammatory cytokines in ANA+ subjects. Table S2. Correlations between the WPI and inflammatory cytokines in ANA+ subjects. [file 13075_2019_2013_MOESM1_ESM.docx]

**Table S1.** Correlations between the FACIT-F score and inflammatory cytokines in ANA^+^ subjects

| Cytokine | All ANA+  ρ | ANS  ρ | UCTD  ρ | SARD  ρ |
| --- | --- | --- | --- | --- |
| 1. No fibromyalgia | | | | |
| Type I IFN | 0.034 | 0.084 | 0.440 (0.089) | -0.160 |
| IL-6 | -0.040 | 0.145 | -0.161 | -0.085 |
| TNF-α | -0.092 | -0.024 | 0.170 | -0.243 |
| 1. Fibromyalgia | | | | |
| Type I IFN | -0.181 | -0.456 (0.072) | 0.226 | 0.134 |
| IL-6 | 0.054 | -0.072 | 0.041 | 0.276 |
| TNF-α | -0.181 | 0.290 | -0.146 | -0.264 |

Significant differences are indicated in bold with the *p* value shown in the brackets. Any *p* values less than 0.1 are also shown as indicative of trends.

**Table S2.**  Correlations between the WPI and inflammatory cytokines in ANA^+^ subjects.

| Cytokine | All ANA+  ρ | ANS  ρ | UCTD  ρ | SARD  ρ |
| --- | --- | --- | --- | --- |
| 1. No fibromyalgia | | | | |
| Type I IFN | 0.031 | -0.090 | -0.126 | 0.244 |
| IL-6 | 0.225 (0.063) | 0.212 | 0.057 | 0.369 (0.058) |
| TNF-α | **0.256 (0.034)** | 0.240 | 0.007 | **0.538 (0.004)** |
| 1. Fibromyalgia | | | | |
| Type I IFN | 0.198 | 0.434 (0.094) | -0.036 | 0.180 |
| IL-6 | 0.196 | 0.061 | 0.041 | 0.276 |
| TNF-α | -0.129 | 0.158 | -0.146 | -0.264 |

Significant differences are indicated in bold with the *p* value shown in the brackets. Any *p* values less than 0.1 are also shown as indicative of trends.
